# Reliable knowledge graph fact prediction via reinforcement learning

**DOI:** 10.1186/s42492-023-00150-7

**Published:** 2023-11-20

**Authors:** Fangfang Zhou, Jiapeng Mi, Beiwen Zhang, Jingcheng Shi, Ran Zhang, Xiaohui Chen, Ying Zhao, Jian Zhang

**Affiliations:** 1 School of Computer Science and Engineering, Central South University, Changsha, Hunan 410083 China; 2 School of Target and Data, Information Engineering University, Zheng Zhou, Henan 450001 China

**Keywords:** Knowledge graph, Fact prediction, Reinforcement learning, Entity heterogeneity, Postwalking mechanism

## Abstract

Knowledge graph (KG) fact prediction aims to complete a KG by determining the truthfulness of predicted triples. Reinforcement learning (RL)-based approaches have been widely used for fact prediction. However, the existing approaches largely suffer from unreliable calculations on rule confidences owing to a limited number of obtained reasoning paths, thereby resulting in unreliable decisions on prediction triples. Hence, we propose a new RL-based approach named EvoPath in this study. EvoPath features a new reward mechanism based on entity heterogeneity, facilitating an agent to obtain effective reasoning paths during random walks. EvoPath also incorporates a new postwalking mechanism to leverage easily overlooked but valuable reasoning paths during RL. Both mechanisms provide sufficient reasoning paths to facilitate the reliable calculations of rule confidences, enabling EvoPath to make precise judgments about the truthfulness of prediction triples. Experiments demonstrate that EvoPath can achieve more accurate fact predictions than existing approaches.

## Introduction

A knowledge graph (KG) structurally organizes facts and knowledge in the form of triples [1]. A triple is expressed as (head entity, relation, tail entity), such as (*Elton_Brand*, *Athlete_plays_in_league*, *NBA*). A KG cannot involve all the facts in an application domain, which is known as incompleteness [2]. Fact prediction is a widely used method to add new facts for a KG [3]. Given that a prediction triple is not involved, its head entity, relation, and tail entity independently exist in a KG. Fact prediction determines whether the prediction triple is true based on the existing triples in the KG. If the result is true, the prediction triple will be added as a new fact into the KG.

**Fig. 1:**
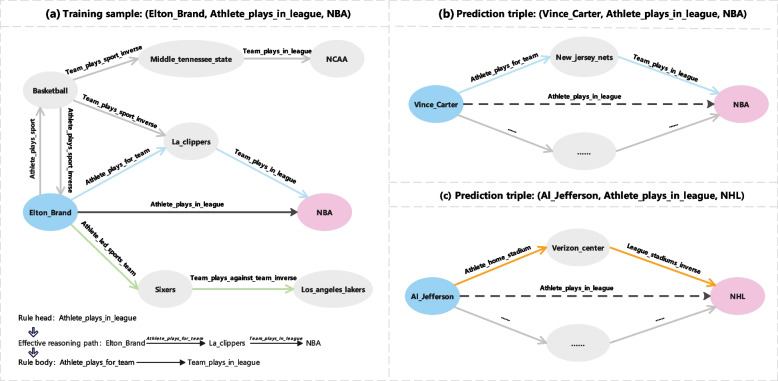
Illustrations of RL-based KG fact prediction. (**a**) A training sample and its partial walking space for an RL-based agent. The black relation is a rule head for rules extracted. The blue path is an effective reasoning path that can extract a rule for fact prediction. The green path is an ineffective reasoning path. (**b**) A prediction triple and its subgraph containing multiple paths linking the head and tail entities of the triple. The blue path matches the rule *Athlete_plays_for_team* $\rightarrow$ *Team_plays_in_league*. Therefore, the prediction relation indicated by the black dashed line is considered true. (**c**) Another prediction triple and its subgraph containing multiple paths. The orange path matches the rule *Athlete_home_stadium* $\rightarrow$ *League_stadiums_inverse*. Therefore, the prediction relation indicated by the black dashed line is considered true. However, this black dashed line relationshipno with walking mechanisms is a false positive

Two-staged reinforcement learning (RL)-based approaches are currently the mainstream methods for fact prediction [4]. The first is a training stage where an RL-based agent conducts a reasoning path-finding process to extract rules for a target relation. All the triples whose relations are the target relation in a KG are used as training facts/samples. Taking the rule *Athlete_plays_in_league* = *Athlete_plays_for_team* $\rightarrow$ *Team_plays_in_league*, *Athlete_plays_in_league* as an example, *Athlete_plays_in_league* is the rule head (i.e., the target relation), and *Athlete_plays_for_team* $\rightarrow$ *Team_plays_in_league* is the ruling body. This rule is extracted based on one or multiple reasoning paths between the head and tail entities of training samples, as illustrated by the blue path in Fig. 1 (a). The agent assigns any rule a confidence value. A high value indicates that the paths corresponding to the rule frequently occur. The second stage is the predicting stage, where the extracted rules are unitized to determine whether a prediction triple is true. First, the agent considers the top *k* (e.g., 3) high-confidence rules for fact prediction. The target relation of these rules equals the relation of the prediction triple. Further, the agent extracts all relation chains within *n* hops (e.g., 50) between the head and tail entities of the prediction triple. Finally, if any chain matches one of these rules, the prediction triple is considered true. Considering the prediction triple in Fig. 1 (b) as an example, a relation chain between *Vince_Carter* and *NBA* (marked in blue) is deemed equal to the rule body of the example mentioned above rule. Thus, the prediction triple is considered true.

However, RL-based approaches often encounter reliability issues of rule confidence, embodying two aspects: (1) some high-confidence rules are not necessarily true in the real world, and (2) some low-confidence rules conform to common sense, resulting in false positives and negatives in prediction, respectively. For example, given a high-confidence rule: *Athlete_plays_in_league* = *Athlete_home_stadium* $\rightarrow$ *League_stadiums_inverse*, indicating that given an athlete playing in a league equals the one playing in a home stadium, the home stadium is used by the league. This rule does not always meet real-world facts because multiple leagues can share a stadium. As shown in Fig. 1 (c), the triple (*Al_Jefferson*, *Athlete_plays_in_league*, *NHL*) would be true using the given high-confidence rule. However, this result is a false positive because *Al_Jefferson* plays in *NBA*. Conversely, given a low-confidence rule: *Team_plays_sport* = *Athlete_plays_for_team_inverse* $\rightarrow$ *Athlete_plays_sport*, indicating that a team playing a sport equals the team having an athlete who is playing the sport. This rule is in line with common sense. However, such a low-confidence rule is probably excluded from the top *k* rules, thereby being underused in fact prediction.

The main reason for the unreliability problem is that the reasoning path-finding process may obtain ineffective reasoning paths. In ineffective reasoning paths, an agent cannot walk from the head entity to the tail entity during a random walk. In this study, we propose a new RL-based approach (“Methods” section) to address the unreliability problem, improving traditional RL-based approaches by providing a new reward mechanism based on entity heterogeneity and a new postwalking mechanism.

Entity heterogeneity is the core factor in obtaining ineffective reasoning paths during a random walk where each step goes from a step-starting entity to a step-ending entity along a step relation. Typically, a step relation (e.g., *Team_plays_sport_inverse* in Fig. 1 (a)) is connected to multiple types of ending entities (e.g., *La_clippers* and *Middle_tennessee_state* in Fig. 1 (a); the two teams belong to different leagues, namely, *NBA* and *NCAA*, respectively), thereby presenting a certain entity heterogeneity. A step-starting entity (e.g., *Basketball* in Fig. 1 (a)) has multiple step relations (e.g., *Team_plays_sport_inverse* and *Athlete_plays_sport_inverse*) with different values of entity heterogeneity. When the random walk selects a step relation with a high entity heterogeneity, the reached step-ending entity presents a significant uncertainty of entity type, causing the random walk to fail in getting the sample’s tail entity.

Based on the above analysis, we propose a new reward mechanism based on entity heterogeneity (“Reward mechanism redesign based on entity heterogeneity” section). For a reasoning path obtained by a random walk, we group the candidate step-ending entities of a walking step into several clusters according to their semantic information using a clustering method. Then, we use the reciprocal of the number of clusters as the value of entity heterogeneity of a walking step. Finally, we multiply the entity heterogeneity values of all steps and add this product as a new reward item into the traditional reward mechanism. This new reward mechanism can guide subsequent random walks to select step relations with relatively low values of entity heterogeneity, thereby improving the probability of obtaining effective reasoning paths.

Ineffective reasoning paths have been obtained for some training samples. Nevertheless, effective reasoning paths exist. As demonstrated in Fig. 1 (a), an ineffective reasoning path (marked in green) that does not reach the tail entity (i.e., *NBA*) of the training sample (*Elton_Brand*, *Athlete_plays_in_league*, *NBA*) has been obtained by a random walk. However, a potentially effective reasoning path (marked in blue) exists between the head and tail entities of the sample. Such potentially effective reasoning paths can provide useful path information for the agent’s walk.

Based on the above analysis, we propose a postwalking mechanism (“Postwalking mechanism design” section) to be triggered for each training sample whose random walk obtains an ineffective reasoning path. We extract all potentially effective reasoning paths for such a training sample and conduct new walks to obtain rewards. The rewards can increase the likelihood of walking on these paths later. This way, the postwalking mechanism can enhance the possibility of obtaining effective reasoning paths in subsequent walks.

We conducted a set of experiments to evaluate the effectiveness of our approach and selected two classic benchmark datasets. We also selected DeepPath [4] as the core reference. We named our approach EvoPath, which stands for the evolution of DeepPath, and selected memoryPath [5] as another core reference including four classic embedding representation-based approaches. The experimental results show that our approach is superior to all the references regarding mean average precision (MAP). The results also demonstrate that EvoPath outperforms DeepPath in terms of Hits@N. The rules and rule confidence values obtained by EvoPath increase the probability of positive samples ranked first or in the top three, further demonstrating the effectiveness of rules with reliable confidence values in fact prediction. Furthermore, we conducted a case study on EvoPath and DeepPath to compare the differences in the rules obtained by the two models. The results demonstrates greater diversity of rules obtained by EvoPath.

In this study, we introduce a new RL-based approach for achieving highly reliable KG fact prediction. We proposed two specific techniques: the reward mechanism based on entity heterogeneity and the postwalking mechanism. Both mechanisms are advantageous for obtaining sufficient reasoning paths during RL model training, thereby extracting reliable rules and the rule confidence values for fact prediction.

## Related works

KG completion tasks can be classified into entity, relation, and fact predictions [3]. Entity, relation, and fact predictions involve predicting another entity given a known entity and relation, represented as (head entity, relation, ?) or (?, relation, tail entity), a relation between two known entities (head or tail entities), and predicting the truth value of a triple given a head entity, a relation, and a tail entity, respectively. It is represented as a prediction triple (head entity, relation, tail entity). Currently, KG fact prediction has three categories of research methods: rule-based, representation-based, and RL-based approaches.

**Rule-based approaches** extract rules from the KG using manual or statistical techniques. Then, they match these obtained rules with paths between the head and tail entities of a prediction triple to perform fact prediction. A successful match indicates that the prediction triple is true. Galárraga et al. [6] proposed an AMIE system in 2013, which efficiently mines rules and matches them with triples in the knowledge base to acquire new facts. In 2016, Cohen [7] introduced TensorLog, which employs a differentiable process for obtaining rules. In the same year, Yang et al. [8] proposed Neural LP, an approach based on TensorLog that enables end-to-end training of logical rules with gradient-based learning. In 2020, Qu et al. [9] introduced RNNLogic, a probabilistic model that trains a rule generator and a reasoning predictor using the EM algorithm. Rule-based approaches are generally accurate and interpretable. However, their effectiveness can be limited by the complexity and scale of KGs.

**Representation-based approaches** map entities and relations to a semantic vector space using a scoring function to calculate the distance between the head-and-tail entities and the relation KG completion tasks. They can be divided into two categories: embedding representation and graph representation techniques. TransE represents the embedding representation techniques [10]. TransE maps the head-and-tail entities and relations into a low-dimensional continuous vector space and uses distance-based score functions to evaluate the authenticity of prediction triples. However, TransE cannot handle 1-to-N and N-to-1 relations.TransH [11], TransR [12], and TransD [13] have been proposed to address this issue. Besides, tensor decomposition models such as RESCAL [14], DistMult [15], ComplEx [16], and ConvE [17] use a similarity-based score function to evaluate the truth of prediction triples. While these embedding representation techniques can effectively capture semantic information about entities and relations in KG, they only use one-hop information and disregard global KG information. However, graph representation techniques can utilize the structural information of multiple hops to capture semantic relationships and contextual information from entities and relations. In 2018, Schlichtkrull et al. [18] first demonstrated that the graph convolutional networks framework can be applied to modeling relational data, specifically for entity prediction and relation prediction tasks. In 2018, Teru et al. [19] proposed a graph neural network (GNN)-based relation prediction framework, GraIL, which reasons over local subgraph structures and has a strong inductive bias to learn entity-independent relational semantics. In 2022, Li et al. [20] proposed CoNR, a new heterogeneous GNN model. In CoNR, entity, and relation representations are mutually updated layer-wise and work together to facilitate downstream tasks. Embedding representation techniques have been used for fact, entity, and relation prediction tasks. Graph representation techniques are mainly applied to entity prediction and relation prediction tasks.

**RL-based approaches** define the reasoning path-finding process in KG as the Markov decision process (MDP), extract rules from the effective reasoning paths obtained from the process, and apply them to fact prediction. Some RL-based approaches are suitable for fact, entity, and relation predictions. In 2019, Lin et al. [21] proposed MultiHop, which introduces reward shaping and action dropout in the path-finding process. In 2021, using a graph attention network, Tiwari et al. [22] proposed DAPath to capture more comprehensive information about neighboring entities and relations. Moreover, it incorporates the GSA mechanism with GRU to consider the memory of relations in the path to guide the agent to walk to the tail entity efficiently. Additionally, some RL-based approaches focus on solving the fact prediction task. DeepPath [4] is the first approach to introduce RL to find reasoning paths combining accuracy, diversity, and efficiency to teach the agent to find effective paths and extract effective rules. The latest approach, MemoryPath proposed by Li et al. [5], is a KG model based on deep RL incorporating LSTM and a graph attention mechanism to form memory components and automatically find promising paths. RL-based approaches can effectively handle the inefficiency of path finding and the lack of explanation in other approaches. With these advantages, RL-based approaches have achieved satisfactory results for fact prediction. Thus, we use RL in our current research on KG fact prediction.

## Results

All of the metrics in this section are covered in detail in the “Methods” section.

We used the classic MAP metric to validate the effectiveness of EvoPath in fact prediction on TransE, TransR, TransH, TransD, DeepPath, MemoryPath, and EvoPath.

**Table 1:** MAP of different models on two datasets

Model	NELL-995	FB15K-237
TransE	0.383	0.277
TransH	0.389	0.309
TransR	0.406	0.302
TransD	0.413	0.303
DeepPath	0.493	0.311
MemoryPath	0.598	0.315
EvoPath	**0.628**	**0.319**

As Table 1 shows, EvoPath improves the MAP values on both datasets. Compared with the widely used embedding representation-based models, our model performs excellently on the NELL-995 dataset, indicating that RL-based models are better suited for fact prediction. Moreover, EvoPath’s fact-prediction ability outperforms the classic DeepPath model and the latest MemoryPath model in the MAP metric, indicating that compared with other models, EvoPath can rank most positive samples highly and most negative samples lowly in test samples, thereby improving the accuracy of fact prediction. EvoPath can discover useful rules that other models have overlooked, thus resulting in more complete rules for each fact prediction task. Furthermore, this result is attributed to the reliability of the rule confidence values, leading to higher scores for positive samples and lower scores for negative samples. Owing to the uneven data distribution in FB15K-237, all models perform poorly on this dataset. However, EvoPath remains the best-performing model.

**Table 2:** Hits@N result of NELL-995

Task	DeepPath		EvoPath	
	Hits@1	Hits@3	Hits@1	Hits@3
*Agent_belongs_to_organization*	0.552	0.655	**0.582**	**0.727**
*Athlete_homestadium*	0.621	0.785	**0.872**	**0.903**
*Athlete_plays_for_team*	0.269	0.432	**0.585**	**0.741**
*Athlete_plays_in_league*	**0.618**	**0.970**	0.603	0.967
*Athlete_plays_sport*	0.800	0.973	**0.877**	**0.977**
*Organization_head_quartered_in_city*	0.863	0.934	**0.899**	**0.947**
*Organization_hired_person*	0.641	0.885	**0.683**	**0.907**
*Person_born_in_location*	0.646	0.708	**0.663**	**0.837**
*Person_leads_organization*	0.644	0.920	**0.655**	**0.925**
*Team_plays_in_league*	0.826	0.919	**0.863**	**0.929**
*Team_plays_sport*	0.490	0.923	**0.644**	**0.952**
*Works_for*	0.550	0.820	**0.597**	**0.890**
Average	0.627	0.827	**0.710**	**0.892**

**Table 3:** Hits@N result of FB15K-237

Task	FB15K-237		EvoPath	
	Hits@1	Hits@3	Hits@1	Hits@3
*ServiceLocation*	0.134	0.537	**0.299**	**0.672**
*FilmDirector*	0.060	0.320	**0.140**	**0.633**
*FilmCountry*	0.275	0.532	**0.526**	**0.788**
*FilmWrittenBy*	0.135	0.480	**0.419**	**0.620**
*CapitalOf*	0.415	0.915	**0.537**	**0.963**
*LocationContains*	0.201	0.506	**0.321**	**0.590**
*MedicineDiseaseRiskFactors*	**0.030**	0.121	0.000	**0.273**
*SymptomOf*	**0.250**	**0.500**	**0.250**	**0.500**
*MusicArtistOrigin*	**0.276**	0.500	**0.276**	**0.517**
*OrganizationLocation*	0.166	0.565	**0.395**	**0.642**
*OrganizationsFounded*	0.023	0.302	**0.163**	**0.372**
*OrganizationMember*	0.032	0.184	**0.119**	**0.346**
*PeopleLanguagesSpoken*	0.140	**0.512**	**0.209**	0.465
*PersonNationality*	0.474	0.776	**0.706**	**0.888**
*BirthPlace*	**0.298**	**0.510**	0.277	0.504
*PeopleProfession*	**0.212**	**0.333**	0.212	0.333
*SportsTeamposition*	**0.017**	**0.483**	0.000	0.450
*TeamSports*	0.522	0.873	**0.873**	**0.943**
*SportsTeamLocation*	**0.259**	**0.482**	**0.259**	**0.482**
*CountryOfOrigin*	0.772	**0.967**	**0.837**	0.957
Average	0.235	0.520	**0.341**	**0.597**

To further demonstrate EvoPath’s effectiveness, we compare it with DeepPath using the Hits@1 and Hits@3 metrics. Hits@N values can directly reflect the model’s performance and more intuitively reflect whether positive samples can be ranked in the top N of the samples. Tables 2 and 3 present both models’ detailed Hits@1 and Hits@3 results on the NELL-995 and FB15K-237 datasets, respectively.

The Hits@1 and Hits@3 metrics focus on the proportion of positive samples ranked first and in the top three, respectively. By comparing Tables 2 and 3, EvoPath outperforms DeepPath overall. On the NELL-995 dataset, DeepPath only outperforms EvoPath for the *Athlet_plays_in_league* task is in two metrics but falls behind EvoPath for other tasks in two metrics. On the FB15K-237 dataset, EvoPath outperforms DeepPath in most fact prediction tasks, demonstrating that EvoPath is more likely to provide correct answers for prediction triples. As EvoPath obtains more effective reasoning paths, the rule data are expected to become more comprehensive. The rule confidence calculation will be more reliable, allowing positive samples to be ranked easily ahead of negative ones with more reliable rule confidence. Furthermore, we observe that EvoPath’s performance on the FB15K-237 dataset is inferior to that on the NELL-995 dataset, attributing to the greater complexity of relations in FB15K-237. Moreover, different relation names may express the same meaning in the dataset. In conclusion, the EvoPath model that we have proposed shows a significant improvement compared with other models in various evaluation metrics. EvoPath can generate more reliable rules, thus resulting in more accurate fact prediction results.

## Discussion

The proposed RL model mainly relies on the relation information from the KG. However, entity information is also important in fact prediction because entities can provide additional contextual information to enhance the model’s performance. For instance, GraIL, which is based on GNN, incorporates entity information by encoding the distance between the head and tail entities of each relationship, enabling the model to learn the structural properties of subgraphs in the KG. So, we intend to explore further how to combine entity information to enhance fact-prediction performance. Besides, this study involves embedding representation techniques, which usually only capture one-hop information about entities and relations. Researchers recently have applied graph representation techniques to entity prediction and relation prediction tasks [18–20]. Graph representation techniques can effectively utilize the structure information of multiple hops to capture semantic relationships and contextual information. Therefore, in future work, we plan to incorporate graph representation techniques to improve the performance of fact prediction.

Second, our model should incorporate an anomaly rule detection mechanism. All models inevitably generate incorrect rules during experimentation. However, our model produces fewer incorrect rules compared with other models. RL is a trial-and-error learning method that cannot guarantee the correctness of the generated rules. If prediction triples match incorrect rules, this scenario may decrease the model’s fact-prediction performance. Therefore, we plan to use methods to identify potential incorrect and anomalous rules. (1) We can employ deep-learning-based anomaly detection methods to identify anomalous rules. For example, convolutional neural networks [23], recurrent neural networks [24], or other models can extract features from rules. Then, the softmax or sigmoid functions can classify rules as normal or abnormal. (2) We can explore using human-machine collaboration for rule anomaly detection. We plan to visualize rule properties, such as entity heterogeneity, using color coding, shape coding, and other graph visualization tools [25–27]. Additionally, we intend to visualize the subgraphs between the head and tail entities of prediction triples together with the rules. This multiperspective KG contextual information will be displayed in a visualization interface [28]. We intend to invite domain experts to participate in human-machine collaborative rule anomaly detection. Through an interactive visualization interface, experts can analyze the anomalies in rules and provide insights, thus obtaining accurate and reliable rules.

Finally, we believe that our model should not only be limited to fact prediction but also be applicable to entity prediction. Fact prediction involves determining the truthfulness of a prediction triple (h, r, t). By contrast, entity prediction involves predicting the tail entity when the head entity and relation are known, e.g., (h, r, ?). Both tasks complete KG under the premise of known relations. Our model can extract reliable rules from the KG to provide reasoning support for each relation. By leveraging these rules and given the head entity, we can perform inference in the KG to obtain the tail entity, thus achieving entity prediction. The highly reliable rules generated by our model can improve the accuracy and efficiency of entity prediction, providing rich and precise technical support for KG completion. For example, it can be applied to specific scenarios such as medical disease diagnosis [29] and malicious behavior analysis of function calls [30].

## Conclusions

In this study, we proposed a new RL-based model called EvoPath for KG fact prediction. The model integrates a reward mechanism based on entity heterogeneity and a postwalking mechanism. The reward mechanism assists an agent in obtaining more effective reasoning paths during a random walk on training samples. By contrast, the postwalking mechanism fully utilizes effective reasoning paths that are ignored. With these two mechanisms, EvoPath accurately calculates the confidence value for each rule by enriching the path information, enhancing the reliability of fact prediction. Compared with mainstream models, the emergence of EvoPath significantly reduces the occurrence of false positives and negatives. Furthermore, it resolves the issue of unreliable rule confidence and strengthens the reliability and accuracy of fact prediction.

## Methods

First, we introduce some notations and describe the task at hand, assume an incomplete KG G={$(e_h,r,e_t)|e_h,e_t\in E,r\in R$} and denote the sets of entities and relations, respectively.

$(e_h,r,e_t)$ represents a fact. Fact prediction determines the truthfulness of a given prediction triple $(e_{h}^{'},r_{t},e_{t}^{'})\notin G$, where $e_{h}^{'}, e_{t}^{'}\in E,r_{t} \in R$. However, $e_{h}^{'}$ and $e_{t}^{'}$ have no direct connection via $r_t$. Instead, some long paths of the form $e_{h}^{'}\overset{r_{1} }{\rightarrow } e_{1}\overset{r_{2} }{\rightarrow } e_{2}...\overset{r_{n} }{\rightarrow } e_{t}^{'}$ from $e_{h}^{'}$ to $e_{t}^{'}$ exist. $e_{i}$ denotes the i-th entity in a path.

RL-based approaches for fact prediction mainly rely on the reasoning path-finding process formulated as an MDP. This process involves performing random walks on samples to identify effective reasoning paths connecting $e_{h}^{'}$ and $e_{t}^{'}$. Then, rules are extracted from these paths and compared with the relation chains of each prediction triple to determine its truthfulness.

One common issue with existing RL-based approaches is the tendency to obtain ineffective reasoning paths during path-finding, resulting in unreliable rule confidence for fact prediction. Our approach is to address this problem, which builds upon the DeepPath model and includes two key improvements: a new reward mechanism based on entity heterogeneity and a postwalking mechanism. Our model aims to identify effective reasoning paths connecting $e_{h}^{'}$ and $e_{t}^{'}$ as much as possible. By increasing the occurrence of effective reasoning paths, the rules extracted from them are assigned with more reliable confidence for fact prediction.

We will first introduce the basic elements of our proposed RL framework, describe the implementation process for the reward and postwalking mechanisms, and present our model’s training method.

### RL framework for KG fact prediction

**Fig. 2:**
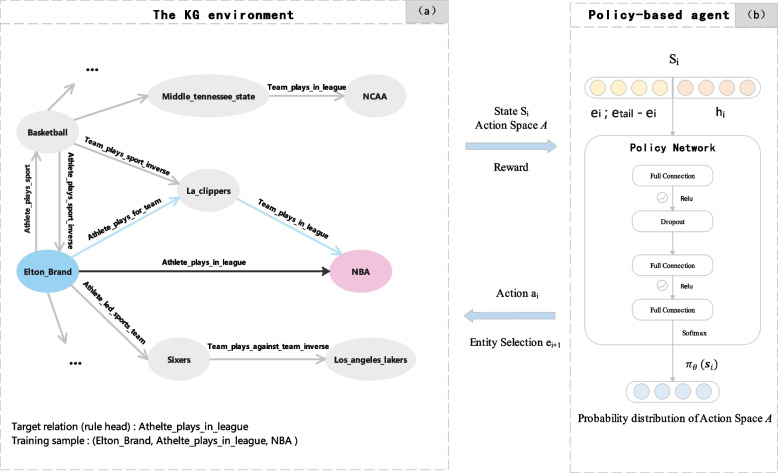
Illustrations of a framework for a KG fact prediction model based on RL. (**a**) The KG environment is modeled as an MDP environment. The black and blue lines represent a target relation trained by RL and a reasoning path obtained by the agent through a random walk, respectively. (**b**) The agent interacts with the MDP environment and takes action based on the policy network to extend the reasoning path

Figure 2 shows that our model comprises the MDP environment and the policy-based agent. The MDP environment refers to the dynamic interaction between the agent and the KG. The policy-based agent utilizes a policy network to determine its selection of specific relations and entities during a random walk in the MDP environment. The agent stops its walk when it meets the termination condition and obtains a reasoning path. The interaction between the policy-based agent and the MDP environment generates basic elements of the RL framework, including state, action, transition, and reward. In the following sections, we introduce these basic elements in the context of KG fact prediction.

**Environment.** In our model, the environment comprises all the relations and entities of a complete KG in a certain domain. This environment remains unchanged throughout the entire training process.

**State.** The state is defined as a vector containing the position information of an agent when the agent walks to the i-th step entity ($e_{i}$) in a KG. In our model, the state consists of entity and historical path information ($h_{i}$). We define the state vector at the i-th step as:

1 $$\begin{aligned} s_{i} =[e_{i},e_{t}-e_{i},h_{i}] \end{aligned}$$

The entity information includes $e_{i}$ and $e_{tail}$, representing the embeddings of the i-th step and tail entities, respectively. To enable the agent to remember the historical path information before the i-th step entity, we utilized a three-layer LSTM network [31]. The LSTM hidden state $h_{i}$ is defined as follows:

2 $$\begin{aligned} h_{0} =\texttt {LSTM}(0,[r_{0},e_{h}])\end{aligned}$$

3 $$\begin{aligned} h_{i} =\texttt {LSTM}(h_{i-1},[a_{i-1},e_{i-1}])\end{aligned}$$

where $r_{0}$ represents a special initial embedding vector and $h_{0}$, the initial hidden state.

**Action.** Our model selects a relation as an action. We define the action space as the set of all relations in the KG, where the action space for each step entity is represented as *A *= *R*. The agent utilizes the policy network to select the most promising relation as an action in the current state. The formula is as follows:

4 $$\begin{aligned} F(a_{i})=\frac{p(a_{i})}{\Sigma _{i=0}^{n}p(a_{i})} ,a_{i}\in A\end{aligned}$$

5 $$\begin{aligned} a_{j}=\texttt {random}(A,F(a_{i})) \end{aligned}$$

where *p(*$a_{i}$*)* represents the probability of action $a_{i}$ output by the policy network, *F(*$a_{i}$*)* represents the normalized probability distribution of action space *A*, and *random()* is a random sampling function.

When the relation taken in the action is not directly connected to the current entity, the other relations are reselected until the selected relation is directly connected to the current entity. Additionally, when the agent reaches the maximum walk length without reaching the tail entity, we use the postwalking mechanism (see “Postwalking mechanism design” section) to guide the agent to take actions that enhance the model’s ability to obtain more effective reasoning paths.

**Transition.** Transition refers to the interaction between the agent and the MDP environment, causing a state change. It is a transition function *P:S*$\times$A$\rightarrow$*S*. The transition of state is achieved by mapping the state of the current state vector to a new state vector. The state transition probability distribution is shown as follows:

6 $$\begin{aligned} s_{i+1} \sim p(s_{i+1} \mid s_{i},a_{i}; \theta )\end{aligned}$$

where $\theta$ denotes the policy network model parameters.

**Reward.** The reward is an indicator of the effectiveness of the actions. We propose a new reward mechanism based on entity heterogeneity to encourage agents to find more effective reasoning paths. Our reward mechanism considers global, path length, and path effectiveness information to build upon a focus on entity heterogeneity. Furthermore, it quantifies the effectiveness of actions into a reward value from multiple dimensions. The reward value is input into the policy network to update its parameters, making it easier for the agent to reach the tail entity in subsequent walk samples. We will detail this reward mechanism in “Reward mechanism redesign based on entity heterogeneity” section.

**Policy network.** The policy network guides an agent forward by taking an action in action space *A*. We used a three-layer fully connected neural network to parameterize the policy function $\pi _{\theta } (a_{i}= r_{i}\mid s_{i})$ mapping the state vector $s_{i}$ to a probability distribution over all possible actions. Furthermore, we added action dropout to block some actions randomly. The output layer is normalized using a softmax function. The policy network $\pi$ is defined as:

7 $$\begin{aligned} \pi _{\theta } ( s_{i})=\texttt {softmax}(W_{3} \texttt {Relu}(W_{2} \texttt {dropout}(\texttt {Relu}(W_{1}s_{i}))))\end{aligned}$$

where $W_1$, $W_2$, and $W_3$ denote the weights.

### Reward mechanism redesign based on entity heterogeneity

Entity heterogeneity is a step relation linked to multiple types of step-ending entities (as shown in “Introduction” section). It is the core factor in obtaining ineffective reasoning paths during a random walk. Previous RL-based approaches did not consider the impact of entity heterogeneity on random walks. Our model quantifies entity heterogeneity into a specific numerical value and incorporates this product into the reward mechanism to upgrade it.

#### Quantify entity heterogeneity reward

Regarding each reasoning path, the entity heterogeneity reward quantifies entity heterogeneity for each relation within the path. The configuration method of the reward is as follows:

1. Entity embedding dimension reduction. For each action the agent takes, we find all the step-ending entities connected to the corresponding step relation and collect them into an entity set. However, high-dimensional embeddings of entities are not conducive to adjusting RL parameters. To address this issue, we used the t-distributed stochastic neighbor embedding [32] dimension reduction technique to map the high-dimensional embedding representation of entities into a two-dimensional space. The reduced dimensional representation vectors will serve as inputs for step 2.
2. Entity clustering. Inspired by Hatem [33], we used the k-nearest neighbors algorithm [34] and knee point detection method to obtain the optimal radius eps value for the current entity set. Then, we used this value as input for the density-based spatial clustering of application with noise [35] clustering algorithm, which outputs *m* clusters of entities and grouped entities with similar semantic features into the same cluster. When the entity set connected to a certain step relation is divided into multiple clusters, we use the number of clusters as a quantitative value to measure the entity heterogeneity of that step relation, denoted as $r_h$= *m*. The $r_h$ metric measures the entity heterogeneity of a given step relation.
3. Entity heterogeneity reward calculation. After a reasoning path *p* is generated, we calculate the entity heterogeneity reward for p using the following formula:

8 $$\begin{aligned} \gamma _{p\_h}=\prod _{i}^{\left| p \right| } \frac{1}{r_{h}^{i} }\end{aligned}$$

where $\left| p\right|$ represents the length of a reasoning path *p*, the number of relations in the reasoning path.

#### Reward mechanism redesign

To encourage the agent to walk autonomously and obtain effective reasoning paths, we proposed a new reward mechanism based on entity heterogeneity, which calculates the reward value for each reasoning path and updates the policy network parameters based on the obtained reward. The mechanism includes the following four scoring criteria:

1. Global reward: If a reasoning path cannot reach the tail entity of a sample, we assign a negative reward to the path to reduce the probability of the agent selecting the relations within that path. Conversely, when a reasoning path reaches a sample’s tail entity, we assign a positive reward to the path to increase the probability of the agent selecting the relations within that path. We represent the last entity of a reasoning path as $e_n$, and the global reward is defined as follows:

9 $$\begin{aligned} \gamma _{gb} =\left\{ \begin{array}{c} +1, if\ e_{n}=e_{t} \\ -0.05,if\ e_{n}\ne e_{t} \end{array}\right. \end{aligned}$$

If $e_n \ne e_{t}$ in a reasoning path, we only use $\gamma _{gb}$ as the reward mechanism.

2) Path length reward: Previous studies show that a short path is more effective in extracting useful rules than a long path [4]. Therefore, to encourage the agent to reach the tail entity in the fewest steps possible, we define the path length reward as:

10 $$\begin{aligned} \gamma _{p\_l} =\frac{1}{\left| p\right| }\end{aligned}$$

3) Path effectiveness reward: Inspired by Li et al. [36], we believe that the semantics of an effective reasoning path should be similar to that of the target relation $r_t$. Therefore, we calculated their semantic similarity as a reward to encourage the agent to walk along an effective reasoning path with a higher semantic similarity with the target relation. The path effectiveness reward is defined as:

11 $$\begin{aligned} \gamma _{p\_e} =\texttt {sim}(P,r_{t})\end{aligned}$$

12 $$\begin{aligned} P=\sum \limits _{i=1}^{n}r_{i}\end{aligned}$$

where *P* represents the embedding representation of the relation chain obtained by sequentially extracting relations from the reasoning path, $r_t$ represents the embedding representation of the target relation, and *sim ()* is the similarity function. We use the cosine similarity as the similarity function.

4) Entity heterogeneity reward: See Quantify entity heterogeneity reward section.

5) Total reward: When the agent obtains a reasoning path through a random walk, we combine the global, path length, path effectiveness, and entity heterogeneity rewards to define the total reward for a reasoning path as follows:

13 $$\begin{aligned} \gamma _{Tt}=\left\{ \begin{array}{c} \lambda _{1}\gamma _{gb}+\lambda _{2}\gamma _{p\_l}+\lambda _{3}\gamma _{p\_e}+\lambda _{4}\gamma _{p\_h},if\ e_{n}=e_{t} \\ \gamma _{gb},if\ e_{n}\ne e_{t} \end{array}\right. \end{aligned}$$

where $\lambda$ is a hyperparameter with $\sum \lambda =1$. In addition, $\gamma _{gb}$ is obtained from Formula (9), $\gamma _{p\_l}$ from Formula (10), $\gamma _{p\_e}$ from Formula (11), and $\gamma _{p\_h}$ from Formula (8).

### Postwalking mechanism design

During the RL training process, some training samples produce ineffective reasoning paths. Nevertheless, effective reasoning paths exist. When these existing effective reasoning paths to provide valid information for training are not used, the agent will have difficulty walking autonomously to reach the tail entity of some samples in the vast walking space.

To extract useful path information for the rule confidence calculation from the training samples that obtained ineffective reasoning paths, we proposed a postwalking mechanism, which will be triggered for each training sample whose random walk obtains an ineffective reasoning path. We show the implementation of this mechanism as follows.

1. **Subgraph extraction**: For each sample that does not obtain an ineffective reasoning path, the head and tail entities of the sample are known. We used the depth-first search algorithm [37] to find a *3-hop* subgraph between the head and tail entities of the sample, containing multiple paths linking the head and tail entities. Then, we sequentially extracted relations on each path and formed a continuous sequence of relations as the path type, i.e.,$type(p)=r_{1}\longrightarrow r_{2}...\longrightarrow r_{n}$.
2. **Path type deduplication**: A subgraph of a sample often has many duplicated path types. The path type with the highest occurrence will affect the reward calculation of those with lower occurrence and greatly impact the policy network. Therefore, we performed a deduplication operation on all the path types obtained from a subgraph to ensure that each is equally calculated once in step 3 for reward calculation.
3. **Force the agent to walk on path types and generate reward**: The model is not guiding the agent to walk in the entire KG based on the policy network. Instead, the agent is forced to walk on each deduplicated path type, thus obtaining an effective reasoning path for each walk. For each agent’s walk, we calculated the corresponding reward to update the policy network’s parameters, increased the probability of selecting the relations in these effective reasoning paths, and enabled the agent to reach the tail entity of the sample autonomously through a random walk in subsequent samples maximally. The reward calculation is as follows:

14 $$\begin{aligned} \gamma _{p\_r}=\frac{1}{3} (\gamma _{p\_l}+\gamma _{p\_e}+\gamma _{p\_h})\end{aligned}$$

where $\gamma _{p\_l}$ is obtained from Formula (10), $\gamma _{p\_e}$ from Formula (11), and $\gamma _{p\_h}$ from Formula (8).

### Training method

To obtain high-reliability rules representing a target relation $r_t$, we trained our model by conducting a random walk on each triple in the training samples for one episode. The specific process of one episode is described as follows:

1. We used a training sample ($e_h$,$r_t$,$e_t$). Starting from its head entity $e_h$($e_0$), when the agent is at the i-th step entity, the MDP environment provides the agent with the state of the entity and the action space *A*. The agent inputs the state and *A* into the policy network $\pi _{\theta }$ ($s_i$) and outputs the probability distribution of the action space *A*.
2. The agent selects the most promising relation from *A* directly connected to the i-th step entity as the action to extend the reasoning path based on the probability distribution. For example, as shown in Fig. 3, if the agent is at $e_1$ and an invalid action $r_1$ that is not directly connected to $e_1$ is selected, the agent will reselect a valid action $r_2$ that is directly connected to $e_1$.

**Fig. 3:**
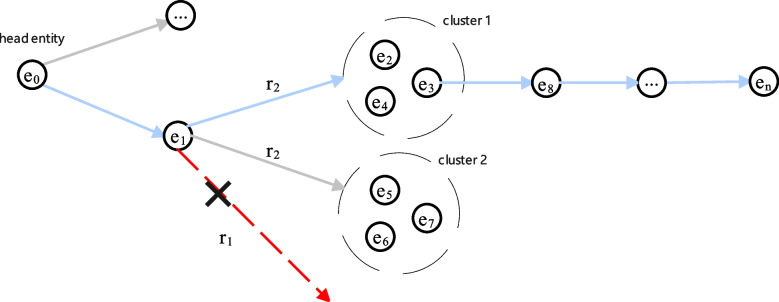
A schematic diagram showing action reselection and entity clustering selection, where the blue solid line represents valid actions, the red dotted line represents invalid action, and multiple entities enclosed by a dotted circle are the results of entity clustering
3. Our model selects the next step entity once a valid action is performed. When selecting a relation as an action, it first uses clustering methods to group entities connected to the relation into different clusters. Then, it calculates the similarity between each cluster’s average and the tail entity’s embedding representations. Finally, the cluster most similar to the tail entity is selected, and an entity from this cluster is randomly selected as the next step entity. As Fig. 3 shows, assuming that the average embedding representation of cluster 1 is more similar to that of the tail entity, our model selects $e_3$ from cluster 1 as the next step entity.
4. After n steps, the agent’s walk terminates at $e_n$. Rewards are computed on the basis of two cases: $e_n$ = $e_t$ and $e_n \ne e_t$. If $e_n$ = $e_t$, the reward is computed directly by the agent using the Formula (13). If $e_n \ne e_t$, after calculating the reward through Formula (13), the postwalking mechanism guides the agent to walk once on each of the path types between the head and tail entities. The reward obtained from each walk is calculated using the Formula (14). Each time a reward is computed, it updates the policy network’s $\theta$ parameter. We use the REINFORCE algorithm [38] and the following policy gradient to update $\theta$:

15 $${\bigtriangledown}_{\theta }J(\theta )\approx {\bigtriangledown}_{\theta} {\sum\limits_{i=1}^{N}} R(s_{N}\mid e_{s},r_{t})log\pi _{\theta }(s_{i})$$

where $\pi _{\theta }$ ($s_i$) represents the probability of the selected action and $R(s_{N}\mid e_{s},r_{t})$ represents the reward obtained after walking with the maximum length *n* from the head entity $e_0$ to the target relation $r_t$. The parameter $\theta$ is updated using an L2-regularized Adam optimizer [39].

Our model only stores this rule during the training process: the agent extracts and stores the rule from an effective reasoning path when it autonomously walks to the tail entity of the sample.

5) After completing all the training, our model will count the times the agent walks each rule and normalize this number to obtain the rule confidence value. The calculation formula is as follows:

16 $$\begin{aligned} \alpha _{i}=\mathbf{conf}\ (\alpha _{i}\mid rule_{i}(r_{t}))=\frac{x_{i}}{{\sum _{i=1}^{N}}x_{i} },\alpha \in [0,1]\end{aligned}$$

where $x_i$ represents the number of times each rule $rule_{i}(r_{t})$ is induced, which is the rule confidence value. Finally, our model generates a descendingly sorted rule set, with rules having confidence values that are ranked higher. The obtained rules and rule confidence values will be used for the KG fact prediction task.

### Experimental settings

#### Dataset

The experiment uses two publicly available benchmark datasets, NELL-995 [40] and FB15K-237 [41]. The NELL-995 dataset has 154213 triples, with 12 types of fact prediction tasks having the same relations within each task, such as *Athlete_plays_for_team*, *Athlete_plays_in_league*. The FB15K-237 dataset has 310116 triples, with 20 fact prediction tasks, such as *capitalOf* and *filmDirector*. Table 4 presents the detailed statistics of the datasets.

**Table 4:** Dataset information

Dataset	Entity	Relation	Triple	Task
NELL-995	75492	200	154213	12
FB15K-237	14505	237	310116	20

We split the triples of each fact prediction task into training and test samples at a ratio of 7:3. The test samples comprised positive and negative samples, where negative samples were generated by randomly replacing the tail entity in positive samples. To enable the agent to reverse the previous action decision during the random walk, we augmented each triple in the dataset with its inverse triple in the form of (t, r_inverse, h).

#### Training and hyperparameters

We obtained the embedding representations of entities and relations used in our model through pretrained TransR, with 100 dimensions for entity and relation embedding vectors. We set the hidden dimension of the LSTM network to 200. The policy network guiding the agent consists of a three-layer fully connected neural network with the ReLU activation function. The first and second layers of the fully connected neural network have 512 and 1024 dimensions, respectively. The output layer has a dimension equal to the number of relations in the KG. Therefore, the output layer dimension is 474 and 400 when using FB15K-237 and NELL-995, respectively.

Additionally, we set the dropout rate for the action dropout mechanism in the policy network to 0.1 for NELL-995 and 0.15 for FB15K-237 datasets. We used the Adam optimizer to update the parameters of the policy network with a learning rate of 0.001 and an L2 regularization of 0.005. The policy network remains unchanged from DeepPath.

Regarding the reward mechanism proposed, we set $\lambda _{1}$ to 0.1, $\lambda _{2}$ to 0.7, $\lambda _{3}$ to 0.1, and $\lambda _{4}$ to 0.1 according to Formula (13). We obtained these parameters through experimental testing and yielded satisfactory experimental results. For each fact prediction task, we trained the model on 300 training samples for 300 episodes, with a maximum walking length of 50 set in each episode.

#### Evaluation

To evaluate the performance of our model and other reference models in KG fact prediction, we utilized the commonly used metric MAP. Additionally, we employ the Hits@N metric to measure the capability of our model and the DeepPath model in ranking positive samples within the top N for each fact prediction task.

The calculation details of each evaluation metric are described below:

- The formula for calculating MAP is as follows:

 

17 $$\begin{aligned} MAP=\frac{1}{t} \sum \limits _{i}^{t}\frac{i}{rank_{i}}\end{aligned}$$

where *t* is the total number of positive samples, *i*/${rank_{i}}$ represents the average precision value of the i-th positive sample, and $rank_{i}$ is the rank of a positive sample in the test samples. A higher MAP value indicates that positive samples are ranked higher in the test samples, indicating better performance in fact prediction.

(b) The formula for calculating Hits@N is as follows:

18 $$\begin{aligned} Hits\;@\;N=\frac{1}{Q}\sum \limits _{i}^{Q}\delta (rank_{i}\le N)\end{aligned}$$

where *Q* is the total number of positive and negative samples with the same head entity, $rank_{i}$ represents the rank of the positive sample among these samples, and $\delta$ is an indicator function that takes a value of 1 if the rank of the positive sample is less than or equal to N and 0 otherwise. In this experiment, we use Hits@1 and Hits@3 as additional metrics to evaluate the model’s performance in fact prediction. A higher Hits@N indicates that positive samples are more likely to be ranked in the top N in the test samples.

#### Baseline and implementation details

Our experiment compared two types of models. The first type was the RL-based model, with our model improving the RL-based DeepPath model. Therefore, we used DeepPath as the baseline model for our experiment. Moreover, to compare the performance of our model with the latest RL-based model, we selected MemoryPath as another RL-based model. The second type is the embedding representation-based model, which adopts a completely different design concept from the RL-based model and is widely used in the fact prediction task. Therefore, we selected TransE, TransR, TransH, and TransD as the comparative models for conducting experiments. We used TransE, TransR, TransH, TransD, DeepPath, MemoryPath, and our improved RL-based model to complete the experiment.

Fact prediction aims to evaluate the truthfulness of a predicted triple. We conducted the implementation of the fact prediction experiment from three aspects. First, we used the widely recognized metric for fact prediction, MAP, to evaluate all models and measure the fact prediction ability of each model. Second, we further compared our model with the baseline model using Hits@1 and Hits@3 metrics to determine which model can more accurately predict the truthfulness of a prediction triple. Finally, we conducted a case study on our model and the baseline model to demonstrate the validity and diversity of the rules learned by our model.

### Case study

We compare the rules used by EvoPath and DeepPath for several fact prediction tasks and analyze the diversity of rules and the effectiveness of rule confidence through a case study. Tables 5 and 6 present the detailed results.

**Table 5:** Comparison of confidence scores for different tasks and rules

Task	Rule	Confidence	
		DeepPath	EvoPath
*Athlete_plays_in_league (NELL-995)*	*Athlete_plays_for_team* $\rightarrow$*Team_plays_in_league*	0.28	**0.46**
	*Athlete_plays_sport* $\rightarrow$*Team_plays_sport_inverse* $\rightarrow$*Team_plays_in_league*	0.26	**0.43**
*Athlete_plays_sport (NELL-995)*	*Athlete_plays_for_team* $\rightarrow$*Team_plays_sport*	0.40	**0.58**
	*Athlete_plays_in_league* $\rightarrow$*Team_plays_in_league_inverse* $\rightarrow$*Team_plays_sport*	0.11	**0.14**
	*Athlete_plays_in_league* $\rightarrow$*League_stadiums* $\rightarrow$*Sport_uses_stadium_inverse*	-	**0.07**
	*Athlete_fly_out_to_sports_team_position* $\rightarrow$*Sport_has_sports_team_position_inverse* $\rightarrow$*Sport_fans_in_country* $\rightarrow$*Sport_fans_in_country_inverse*	**0.08**	-
*filmWrittenBy (FB15K-237)*	*/award/award_nominee/award_nominations./award/award_nomination/nominated_for_inverse*	0.33	**0.47**
	*/film/actor/film./film/performance/film_inverse*	**0.33**	0.20
	*/film/director/film_inverse*	**0.17**	0.09
	*/film/film/cinematography_inverse*	**0.17**	0.05

**Table 6:** Rule count

Target relation	Model	Rule count	Length = 1	Length = 2	Length = 3	Length > 3
*Athlete_plays_in_league*	DeepPath	24	0	3	6	15
	EvoPath	31	1	2	5	23
*Athlete_plays_sport*	DeepPath	21	0	3	4	14
	EvoPath	40	0	3	6	31
*filmWrittenBy*	DeepPath	4	4	0	0	0
	EvoPath	37	6	3	2	26

The experimental results demonstrate the effectiveness of EvoPath in obtaining rules from three aspects as follows. (1) EvoPath is more likely than DeepPath to extract reliable rules. For instance, for the relation *Athlete_plays_sport*, EvoPath can extract a reliable rule: *Athlete_plays_in_league*$\rightarrow$*League_stadiums*$\rightarrow$*Sport_uses_stadium_inverse*, while DeepPath cannot. (2) The rules extracted by EvoPath have fewer high-entity heterogeneity relations, while the rules induced by DeepPath have multiple ones. For instance, *Athlete_fly_out_to_sports_team_position*$\rightarrow$*Sport_has_sports_team_position_inverse*$\rightarrow$*Sport_fans_in_country*$\rightarrow$*Sport_fans_in_country_inverse* is extracted by DeepPath but not by EvoPath because EvoPath considers such rules with high-entity heterogeneity relations unreliable. (3) The reliable rules extracted by EvoPath have higher confidence than those extracted by DeepPath. For instance, for the rule *Athlete_plays_for_team*$\rightarrow$*Team_plays_in_league*, EvoPath gives it a confidence of 0.18 higher than DeepPath. Conversely, the unreliable rules extracted by EvoPath have lower confidence than those extracted by DeepPath. For instance, the rule */film/director/film_inverse* means a director directs a movie. However, the rule head *filmWrittenBy* means a person writes a movie. Although both models extract this rule, EvoPath’s confidence is 0.08 lower than that of DeepPath.

Table 6 shows the number of rules extracted by DeepPath and EvoPath on different lengths. The results indicate that EvoPath obtains more diverse rules than DeepPath by learning short and long paths, which are useful for predicting triples that lack short paths. For instance, EvoPath learns 17 additional rules with a length > 3 for the target relation *Athlete_plays_sport* compared with DeepPath. This feature can be attributed to the postwalking mechanism in EvoPath, helping the agent accurately reach the tail entity when expanding long paths. Additionally, EvoPath extracts more rules for the same target relation than DeepPath because the postwalking and reward mechanisms help the agent find more effective reasoning paths.

## Data Availability

The NELL-995 dataset and FB15K-237 dataset are available at https://github.com/shehzaadzd/MINERVA/tree/master/datasets/data_preprocessed.

## Abbreviations

KG: Knowledge graph
RL: Reinforcement learning
MAP: Mean average precision
MDP: Markov decision process
GNN: Graph neural network
